# Expanded A-DROP Score: A New Scoring System for the Prediction of Mortality in Hospitalized Patients with Community-acquired Pneumonia

**DOI:** 10.1038/s41598-018-32750-2

**Published:** 2018-10-01

**Authors:** June Hong Ahn, Eun Young Choi

**Affiliations:** Department of Internal Medicine, Yeungnam University Medical Center, College of Medicine, Yeungnam University, Daegu, South Korea

## Abstract

There are several established prognostic scoring systems for community-acquired pneumonia (CAP). The Pneumonia Severity Index (PSI) is a prediction rule consisting of 20 variables to identify low-risk patients with CAP. Although PSI had high discrimination ability, it is complex to calculate and difficult to use in busy hospital settings. The CURB-65 score is much simpler to use than is PSI, but it has lower sensitivity for predicting mortality compared with PSI. The A-DROP score is a modified version of the CURB-65 score and provides similar predictive power to that of CURB-65. This study was performed to determine whether a simpler score (CURB-65, A-DROP), expanded with a small number of additional variables, can predict mortality more accurately than PSI. We conducted a retrospective observational study of 1,031 patients with CAP who were hospitalized at a tertiary teaching hospital. We used age, sex, comorbidities, vital signs, and laboratory findings as prognostic variables. We compared the PSI, CURB-65, and A-DROP scores using receiver operating characteristic curve analysis. The areas under the curves (AUCs) of PSI, CURB-65, and A-DROP were 0.735, 0.701, and 0.730, respectively.Multivariable analysis identified malignancy [odds ratio (OR): 2.17, 95% confidence interval (CI): 1.13–4.17], respiration rate ≥ 24/min [OR: 2.18, 95% CI: 1.24–3.82], heart rate ≥ 100/min [OR: 2.92, 95% CI: 1.68–5.08], albumin ≤ 3.09 g/dL [OR: 3.85, 95% CI: 2.09–7.07], lactate > 1.7 mmol/L [OR: 2.59, 95% CI: 1.53–4.38], and N-terminal prohormone brain natriuretic peptide > 500 pg/mL [OR: 2.23, 95% CI: 1.26–3.95] as prognostic factors. Using the prognostic variables identified in the multivariable analysis, we assembled a new scoring system, the expanded A-DROP score. The AUC of this score for the prediction of 28-day mortality was 0.834 (95% CI: 0.794–0.874). Bootstrap validation yielded an estimated AUC of 0.833, indicating negligible overfitting of the model.The expanded A-DROP score is a relatively simple and effective scoring system, and its predictive value was superior to those of other scoring systems.

## Introduction

Community-acquired pneumonia (CAP) is a significant cause of morbidity and mortality throughout the world. It is the seventh-leading cause of death in the United States, where about 910,000 episodes of CAP occur annually in adults aged >65 years^1^. In Korea, CAP ranked tenth (7.1 deaths per 100,000 population) among all causes of death in 2004 and fifth (21.4 deaths per 100,000 population) in 2013, which was the highest mortality rate among causes of death due to infection^2^. In the management of CAP, initial assessment of disease severity is essential because it determines the therapeutic approach, including decisions about the need for hospitalization or intensive care unit admission, the extent of diagnostic testing, and the type of antibiotic treatment^1^.

Several established severity scores and multiple biomarkers have been used to assess the severity of CAP. The Pneumonia Severity Index (PSI) is a predictive tool used to identify low-risk patients with CAP. Proposed in 1997, the PSI consists of 20 variables, including demographic characteristics, comorbidities, and physical examination, laboratory, and radiographic findings. Although the PSI has high discriminatory value, its calculation is complex, it is difficult to use in busy hospital settings, and it underestimates disease severity in young patients with no comorbidity^3^.

The CURB-65 score, consisting of confusion, urea >7 mmol/L, respiratory rate ≥30 breaths/min, blood pressure (systolic <90 mmHg or diastolic ≤60 mmHg), and age ≥65 years was proposed in 2003. It is simple to use, but was developed to identify patients with severe CAP at high risk of mortality. Thus, it does not identify patients at low risk of mortality, for whom home treatment might be suitable^4^.

The A-DROP score, consisting of age ≥70 years in males or ≥75 years in females, blood urea nitrogen ≥21 mg/dL or dehydration, oxyhemoglobin saturation measured by pulse oximetry ≤90% or partial oxygen pressure in arterial blood ≤60 mmHg, confusion, and systolic blood pressure ≤90 mmHg, is a modified version of the CURB-65 score proposed by the Japanese Respiratory Society in 2006^5^. Its predictive power is similar to that of the CURB-65 and PSI^6,7^.

This study was conducted to identify prognostic factors for 28-day mortality in patients with CAP, and to compare the predictive value of three pneumonia severity scores. Following these analyses, we developed a simpler and more accurate scoring system by expanding the A-DROP score, and evaluated its efficacy compared with that of preexisting scores for severity assessment.

## Methods

### Study design

We performed a retrospective observational study of 1,031 patients with CAP who were hospitalized at Yeungnam University Hospital (a 930-bed, university-affiliated, tertiary referral hospital in Daegu, South Korea) between March 2012 and February 2014.

During the study period, all consecutive CAP patients admitted to the hospital via the emergency or outpatient department were considered to be eligible for inclusion. Pneumonia was defined as the presence of new radiographic infiltrate and at least two of the following criteria: fever (>38 °C) or hypothermia (≤35 °C), new cough with or without sputum production, pleuritic chest pain, dyspnea, and altered breath sounds on auscultation^8^. This study included some patients previously defined as having healthcare-associated pneumonia (HCAP), because the 2016 American Thoracic Society and Infectious Diseases Society of America (ATS/IDSA) guidelines removed the concept of HCAP^9^. Therefore, we considered previously defined HCAP as CAP in this study. This study excluded patients with hospital-acquired pneumonia that developed after >48 h hospitalization, those aged <18 years, immunocompromised patients (such as those with neutropenia after chemotherapy, human immunodeficiency virus infection, solid organ transplant recipients, and those receiving corticosteroids or other immunosuppressive agents), and patients with active *Mycobacteria tuberculosis* infection. This study only included the first admission in the case of multiple admissions for a same patient. The primary endpoint was 28-day mortality, and several clinical variables were compared between the survival and non-survival groups.

Antibiotic therapy was initiated according to the 2007 ATS/IDSA guidelines^1^, according to the attending physicians’ decisions and taking into consideration the severity of the disease and patients’ underlying conditions. When a pathogen was identified, antibiotic therapy was modulated according to the susceptibility test results.

As reported previously^10^, identified pathogens that were not susceptible to β-lactams, macrolides, and fluoroquinolones were defined as multidrug-resistant (MDR) pathogens.

This study was conducted in accordance with the Declaration of Helsinki. It was reviewed and approved by the institutional review board of our hospital (YUMC IRB 2017-11-013), and the requirement for informed consent was waived because of the retrospective design.

### Data collection

Data on patients’ age, sex, comorbidities, and vital signs were collected. The severity of pneumonia was assessed in all patients on admission using the PSI^3^, CURB-65 score [confusion, urea >7 mmol/L, respiratory rate ≥30 breaths/min, blood pressure (systolic <90 mmHg or diastolic ≤60 mmHg), and age ≥65 years]^4^, and A-DROP score [age ≥70 years for males and ≥75 years for females, blood urea nitrogen (BUN) ≥21 mg/dL or dehydration, oxyhemoglobin saturation measured by pulse oximetry ≤90% or partial pressure of oxygen in arterial blood (PaO_2_) ≤60 mmHg, confusion, and systolic blood pressure ≤90 mmHg]^5^.

Laboratory results, including complete blood counts with differentials and C-reactive protein (CRP), procalcitonin, lactate, N-terminal prohormone brain natriuretic peptide (NT-ProBNP), BUN, creatinine, albumin, glucose, sodium, and alkaline phosphatase (ALP) levels, were reviewed. PaO_2_, partial pressure of carbon dioxide in arterial blood (PaCO_2_), fraction of inspired oxygen (FiO_2_) and PaO_2_/FiO_2_ values were obtained from the patients’ arterial blood samples. The laboratory findings were analyzed within 24 hours after admission.

Two investigators (JHA and EYC) independently reviewed the baseline data using electronic medical records.

### Statistical analysis

Continuous variables are expressed as means ± standard deviation and were compared using Student’s *t*-test or the Mann–Whitney *U* test. Categorical variables were compared using the chi-squared test or Fisher’s exact test. Receiver operating characteristic (ROC) curve analyses were performed to assess the effectiveness of pneumonia severity scores for predicting CAP prognosis. When continuous variables were converted to categorical variables, cut-off values were determined using ROC curves. Multivariable logistic regression analyses were performed to identify independent prognostic factors for mortality using variables with *p* values < 0.05 in univariable analyses, with odds ratios (ORs) and 95% confidence intervals (CIs). In all analyses, two-tailed *p* values < 0.05 were considered to indicate significance.

We conducted internal validation of the expanded A-DROP model to address the possibility of overfitting causing optimism regarding the model’s performance. Among several internal validation methods, bootstrapping is known to provide more stable estimates with lower bias than other methods^11^. We used the bootstrap resampling method whereby our derived model was repeatedly fit in 1,000 bootstrap samples. The AUC estimates of these bootstrap models in the original study sample were then computed. We took the mean AUC for these 1,000 bootstrap models to represent the estimate of model performance. All statistical procedures were performed using SPSS software (ver. 21.0; SPSS Inc., Chicago, IL, USA) and R statistical software (version 3.4.3, Vienna, Austria).

## Results

### Baseline characteristics

During the study period, 1,046 patients with CAP were hospitalized. After application of the exclusion criteria, 1,031 patients were enrolled in the study. Enrolled patients were divided into two groups: 935 (90.7%) survivors and 96 (9.3%) non-survivors.

The demographic and baseline clinical characteristics of the patients with CAP are presented in Table 1. The non-survival group was significantly older than the survival group (mean age: 73.7 ± 11.2 vs. 68.7 ± 14.5 years, *p* < 0.001). The non-survival group was significantly more likely to have comorbidities, such as congestive heart failure (14.6% vs. 7.1%, *p* = 0.009), dementia (13.5% vs. 7.2%, *p* = 0.026) and malignancy (19.8% vs. 10.4%, *p* = 0.005).

**Table 1:** Baseline characteristics of the study patients.

	Survival (n = 935)	Non-survival (n = 96)	P value	Whole population
28-day mortality				9.3%
Mean age (years)	68.7 ± 14.5	73.7 ± 11.2	0.001	69.2 ± 14.3
Male, n (%)	617 (66.0%)	71 (74.0%)	0.115	688 (66.7%)
**Smoking status**, **n (%)**				
Current	150 (16.0%)	16 (16.7%)	0.349	166 (16.1%)
Ex-smoker	163 (17.4%)	19 (19.8%)		182 (17.7%)
Never-smoker	622 (66.5%)	61 (63.5%)		683 (66.2%)
Pack-years	12.0 ± 20.9	16.1 ± 32.4	0.390	12.4 ± 22.2
**Comorbidities**, **n (%)**				
Myocardial infarction	33 (3.5%)	3 (3.1%)	1.000	36 (3.5%)
Congestive heart failure	66 (7.1%)	14 (14.6%)	0.009	80 (7.8%)
Peripheral vascular disease	74 (7.9%)	13 (13.5%)	0.059	87 (8.4%)
Cerebrovascular disease	179 (19.1%)	25 (26.0%)	0.106	204 (19.8%)
Dementia	67 (7.2%)	13 (13.5%)	0.026	80 (7.8%)
Chronic pulmonary disease^a^	291 (31.1%)	29 (30.2%)	0.854	320 (31.0%)
Connective tissue disease	17 (1.8%)	3 (3.1%)	0.422	20 (1.9%)
Mild liver disease	14 (1.5%)	1 (1.0%)	1.000	15 (1.5%)
Diabetes mellitus	198 (21.2%)	23 (24.0%)	0.527	221(21.4%)
Renal disease	35 (3.7%)	5 (5.2%)	0.411	40 (3.9%)
Malignancy^b^	97 (10.4%)	19 (19.8%)	0.005	116 (11.3%)
Moderate to severe liver disease	16 (1.7%)	3 (3.1%)	0.327	19 (1.8%)
Mechanical ventilator, n (%)	51 (5.5%)	18 (18.8%)	<0.001	69 (6.7%)
ICU admission, n (%)	43 (4.6%)	15 (15.6%)	<0.001	58 (5.6%)
LOS	12.7 ± 13.7	8.9 ± 10.3	0.008	12.3 ± 13.2
MDR pathogen, n (%)	87 (9.3%)	17 (17.7%)	0.009	104 (10.1%)
CURB-65	1.5 ± 1.1	2.3 ± 1.1	<0.001	1.6 ± 1.1
CURB-65 (≥3)	165 (17.6%)	40 (41.7%)	<0.001	205 (19.9%)
PSI class	3.5 ± 1.1	4.2 ± 0.9	<0.001	3.6 ± 1.1
PSI class V	139 (14.9%)	38 (39.6%)	<0.001	177 (17.2%)

Data are expressed as mean ± SD for continuous variables.

^a^Chronic lung disease includes chronic obstructive pulmonary disease, asthma, bronchiectasis, and interstitial lung disease.

^b^Malignancy includes cancer that was active at the time of admission or was diagnosed within one year of admission.

ICU: intensive care unit; LOS: length of stay; MDR: multidrug-resistant; PSI: Pneumonia Severity Index.

CURB-65, PSI, and A-DROP scores differed significantly between the survival and non-survival groups. Table 2 presents 28-day mortality rates according to these three scores. A significant increase in mortality was observed with higher CURB-65, PSI, and A-DROP scores.

**Table 2:** Severity risk classification of the study patients.

	Survival (n = 935)	Non-survival (n = 96)	Mortality rate	Subgroup mortality	Pvalue
**CURB-65**, **n (%)**					
0	153 (16.4%)	4 (4.2%)	2.5%	3.8% (0–1)	<0.001
1	349 (37.3%)	16 (16.7%)	4.4%		
2	268 (28.7%)	36 (37.5%)	11.8%	11.8% (2)	
3	128 (13.7%)	27 (28.1%)	17.4%	19.5% (3–5)	
4	33 (3.5%)	10 (10.4%)	23.3%		
5	4 (0.4%)	3 (3.1%)	42.9%		
**PSI grade**, **n (%)**					
I	78 (8.3%)	3 (3.1%)	3.7%	2.6% (I–III)	<0.001
II	77 (8.2%)	1 (1.0%)	1.3%		
III	176 (18.8%)	5 (5.2%)	2.8%		
IV	465 (49.7%)	49 (51.0%)	9.5%	9.5% (IV)	
V	139 (14.9%)	38 (39.6%)	21.5%	21.5% (V)	
**A-DROP**, **n (%)**					
0	248 (26.5%)	6 (6.3%)	2.4%	3.9% (0–1)	<0.001
1	342 (36.6%)	18 (18.8%)	5.0%		
2	232 (24.8%)	37 (38.5%)	13.8%	13.8% (2)	
3	85 (9.1%)	20 (20.8%)	19.0%	23.6% (3–5)	
4	26 (2.8%)	14 (14.6%)	35.0%		
5	2 (0.2%)	1 (1.0%)	33.3%		

PSI: Pneumonia Severity Index.

The clinical and laboratory findings are shown in Table 3. Of the vital signs, systolic blood pressure was significantly lower in the non-survival group (114.4 ± 26.8 mmHg vs. 120.5 ± 23.4 mmHg, p = 0.047). Respiration rate and heart rate were significantly higher in the non-survival group (26.6 ± 5.7vs. 22.7 ± 4.6, p < 0.001; and 107.6 ± 22.2 vs. 93.0 ± 19.4, p < 0.001).

**Table 3:** Initial clinical and laboratory parameters.

	Survival (n = 935)	n	Non-survival (n = 96)	n	P value
Systolic BP (mmHg)	120.5 ± 23.4	934	114.4 ± 26.8	96	0.047
Diastolic BP (mmHg)	72.9 ± 14.5	934	68.9 ± 16.6	96	0.083
Body temperature (°C)	37.4 ± 0.8	935	37.5 ± 1.0	96	0.737
Respiratory rate (breaths/min)	22.7 ± 4.6	935	26.6 ± 5.7	96	<0.001
Heart rate (beats/min)	93.0 ± 19.4	935	107.6 ± 22.2	96	<0.001
PH	7.43 ± 0.7	932	7.39 ± 0.1	96	0.001
PaCO_2_ (mmHg)	36.9 ± 20.6	932	35.9 ± 12.3	95	0.642
PaO_2_ (mmHg)	70.8 ± 21.9	935	65.3 ± 22.2	96	0.020
PaO_2_/FiO_2_ ratio	318.2 ± 88.4	935	268.4 ± 77.1	96	<0.001
Lactate (mmol/L)	1.6 ± 1.1	915	2.9 ± 2.2	94	<0.001
BUN (mg/dL)	17.6 ± 12.2	935	28.5 ± 20.8	96	<0.001
Creatinine (mg/dL)	1.2 ± 0.8	935	1.6 ± 1.2	96	<0.001
Albumin (g/dL)	3.3 ± 0.7	935	2.7 ± 0.6	96	<0.001
Sodium (mEq/L)	136.8 ± 5.0	935	136.0 ± 7.5	96	0.041
Glucose (mg/dL)	158.0 ± 74.6	736	183.8 ± 141.7	91	0.262
White blood cells (×10^3^/µL)	11.5 ± 6.2	935	11.8 ± 7.3	96	0.928
Hemoglobin (g/dL)	12.3 ± 2.0	935	11.3 ± 2.1	96	<0.001
Hematocrit (%)	36.1 ± 5.9	931	33.6 ± 6.0	96	<0.001
Platelet (×10^3^/µL)	283.1 ± 118.7	934	257.4 ± 125.2	96	0.045
Alkaline phosphatase (IU/L)	210.6 ± 114.6	902	251.1 ± 170.8	92	0.010
Procalcitonin (ng/mL)	3.4 ± 16.4	917	10.9 ± 34.0	95	<0.001
C-reactive protein (mg/dL)	10.9 ± 10.1	932	16.7 ± 11.5	95	<0.001
NT-ProBNP (pg/mL)	1152.2 ± 2959	902	4857.5 ± 7771	90	<0.001
Pleural effusion	152 (16.3%)	935	24 (25.0%)	96	0.030

Data are expressed as mean ± SD for continuous variables.

BP: blood pressure; PaCO_2_: partial pressure of arterial carbon dioxide; PaO_2_: partial pressure of arterial oxygen; FiO_2_: fraction of inspired oxygen; BUN: blood urea nitrogen; NT-ProBNP: N-terminal pro-brain natriuretic peptide.

With regard to the laboratory findings, arterial pH, PaO_2_, PaO_2_/FiO_2_, lactate, BUN, creatinine, albumin, sodium, hemoglobin, hematocrit, platelet, ALP, procalcitonin, CRP, and NT-ProBNP differed significantly between the two groups. PaCO_2_, glucose level, and white blood cell count were not significantly different between the two groups.

### Prognostic factors for 28-day mortality in patients with CAP

In univariable analysis, age, pleural effusion, comorbidities (including congestive heart failure, dementia, and malignancy), vital signs (including systolic blood pressure, respiratory and heart rates), and laboratory findings (including arterial pH, hematocrit, platelet count, and PaO_2_, PaO_2_/FiO_2_, BUN, creatinine, albumin, hemoglobin, ALP, CRP, lactate, procalcitonin, and NT-ProBNP levels) were significant prognostic factors for mortality in patients with CAP. In multivariable analysis, malignancy (OR: 1.99, 95% CI: 1.04–3.81, *p* = 0.039), respiratory rate (OR: 1.06, 95% CI: 1.02–1.10, *p* = 0.008), heart rate (OR: 1.02, 95% CI: 1.01–1.03, *p* < 0.001), albumin level (OR: 0.27, 95% CI: 0.18–0.41, *p* < 0.001), platelet count (OR: 0.998, 95% CI 0.996–1.00, *p* = 0.03), lactate level (OR: 1.28, 95% CI: 1.10–1.49, *p* = 0.002) and NT-ProBNP level (OR: 1.00, 95% CI: 1.00–1.00, *p* = 0.001) were significant prognostic factors (Table 4).

**Table 4:** Univariable and multivariable analysis of prognostic factors for 28-day mortality.

Prognostic factors	Univariable			Multivariable		
	OR	95% CI	P value	OR	95% CI	P value
Age	1.03	1.01–1.05	0.001			
Pleural effusion	1.72	1.05–2.81	0.032			
Congestive heart failure	2.25	1.21–4.18	0.010			
Dementia	2.03	1.08–3.83	0.029			
Malignancy	2.13	1.24–3.67	0.006	1.99	1.04–3.81	0.039
Systolic BP	0.99	0.98–1.00	0.016			
Respiratory rate (beats/min)	1.13	1.09–1.17	<0.001	1.06	1.02–1.10	0.008
Heart rate (beats/min)	1.03	1.02–1.04	<0.001	1.02	1.01–1.03	<0.001
PH	0.008	0.001–0.07	<0.001			
PaO_2_ (mmHg)	0.99	0.98–1.00	0.017			
PaO_2_/FiO_2_ ratio	0.99	0.99–1.00	<0.001			
BUN (mg/dL)	1.04	1.03–1.05	<0.001			
Creatinine (mg/dL)	1.44	1.20–1.73	<0.001			
Albumin (g/dL)	0.27	0.19–0.38	<0.001	0.27	0.18–0.41	<0.001
Hemoglobin (g/dL)	0.78	0.70–0.87	<0.001			
Hematocrit (%)	0.93	0.89–0.96	<0.001			
Platelet (×10^3^/µL)	0.998	0.996–1.00	0.046	0.998	0.996–1.00	0.030
Alkaline phosphatase (IU/L)	1.002	1.001–1.003	0.004			
C-reactive protein (mg/dL)	1.05	1.03–1.07	<0.001			
Lactate (mmol/L)	1.52	1.35–1.71	<0.001	1.28	1.10–1.49	0.002
Procalcitonin (ng/mL)	1.01	1.00–1.02	0.001			
NT-ProBNP (pg/mL)	1.00	1.00–1.00	<0.001	1.00	1.00–1.00	0.001

OR: odds ratio; CI: confidence interval; BP: blood pressure; PaO_2_: partial pressure of arterial oxygen; FiO_2_: fraction of inspired oxygen; BUN: blood urea nitrogen; NT-ProBNP: N-terminal pro-brain natriuretic peptide.

When the above-listed significant variables were converted to categorical variables using cut-off values, malignancy (OR: 2.17, 95% CI: 1.13–4.17, *p* = 0.021), respiratory rate ≥24 breaths/min (OR: 2.18, 95% CI: 1.24–3.82, *p* = 0.007), heart rate ≥100 beats/min (OR: 2.92, 95% CI: 1.68–5.08, *p* < 0.001), albumin ≤3.09 g/dL (OR: 3.85, 95% CI: 2.09–7.07, *p* < 0.001), lactate >1.7 mmol/L (OR: 2.59, 95% CI: 1.53–4.38, *p* < 0.001) and NT-ProBNP >500 pg/mL (OR: 2.23, 95% CI: 1.26–3.95, *p* = 0.006) were significant prognostic factors in patients with CAP (Table 5). Table 6 shows the prevalence of these risk factors in different classes of the CURB-65 score, PSI, and A-DROP scores.

**Table 5:** Multivariable analysis of prognostic factors for 28-day mortality using categorical variables.

	OR	95% CI	P value
Malignancy	2.17	1.13–4.17	0.021
Respiratory rate ≥24 breaths/min	2.18	1.24–3.82	0.007
Heart rate ≥100 beats/min	2.92	1.68–5.08	<0.001
Albumin ≤3.09 g/dL	3.85	2.09–7.07	<0.001
Lactate >1.7 mmol/L	2.59	1.53–4.38	<0.001
NT-ProBNP >500 pg/mL	2.23	1.26–3.95	0.006

OR: odds ratio; CI: confidence interval; NT-ProBNP: N-terminal pro-brain natriuretic peptide.

**Table 6:** The prevalence of risk factors detected in the different classes of CURB-65 score, PSI and A-DROP scores in CAP patients.

Subgroup	Patients	Malignancy	Respiratory rate ≥24/min	Heart rate ≥100/min	Albumin ≤3.09 g/dL	Lactate >1.7 mmol/L	NT-ProBNP >500 pg/mL
**CURB-65**, **n (%)**							
0–1	522 (50.6%)	49 (9.4%)	125 (23.9%)	164 (31.4%)	158 (30.3%)	126 (24.1%)	99 (19.0%)
2	304 (29.5%)	43 (14.1%)	119 (39.1%)	101 (33.2%)	156 (51.3%)	90 (29.6%)	139 (45.7%)
3–5	205 (19.9%)	24 (11.7%)	137 (66.8%)	118 (57.6%)	124 (60.5%)	112 (54.6%)	134 (65.4%)
**PSI grade**, **n (%)**							
I-III	340 (33.0%)	19 (5.6%)	73 (21.5%)	95 (27.9%)	72 (21.2%)	58 (17.1%)	42 (12.4%)
IV	514 (49.9%)	70 (7.8%)	203 (39.5%)	193 (37.5%)	248 (48.2%)	178 (34.6%)	206 (40.1%)
V	177 (17.2%)	27 (15.3%)	105 (59.3%)	95 (53.7%)	118 (66.7%)	92 (52.0%)	124 (70.1%)
**A-DROP**, **n (%)**							
0–1	614 (59.6%)	58 (9.4%)	181 (29.5%)	192 (31.2%)	205 (33.4%)	146 (23.8%)	133 (21.7%)
2	269 (26.1%)	42 (15.6%)	118 (43.9%)	112 (41.6%)	129 (48.0%)	102 (37.9%)	138 (51.3%)
3–5	148 (14.4%)	16 (10.8%)	82 (55.4%)	79 (53.4%)	104 (70.3%)	80 (54.1%)	101 (68.2%)

PSI: Pneumonia Severity Index; CAP: community-acquired pneumonia.

### ROC curves for mortality prediction with the three preexisting pneumonia severity scoring systems

**Figure 1:**
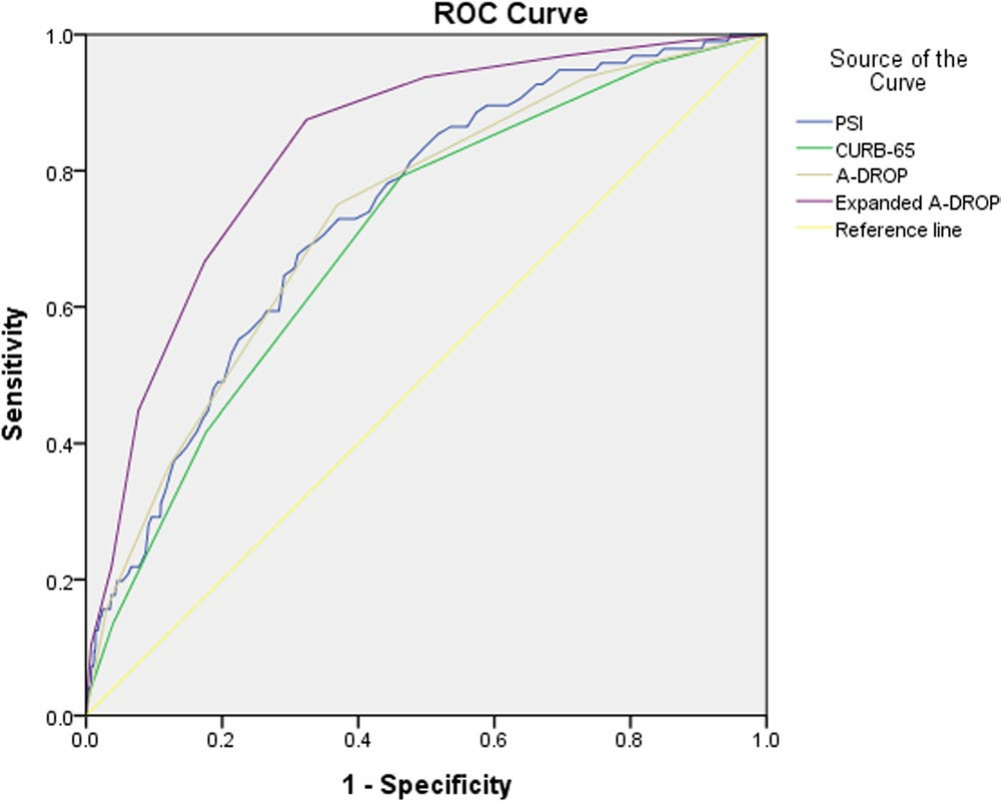
ROC curves for scoring systems in the study patients. ROC: Receiver operating characteristic.

ROC analysis generated areas under the curve (AUCs) for the prediction of 28-day mortality of 0.735 (95% CI: 0.686–0.784), 0.701 (95% CI: 0.648–0.754), and 0.730 (95% CI: 0.678–0.782) for the PSI, CURB-65, and A-DROP scores, respectively (Fig. 1).

### New scoring system developed for assessment of pneumonia severity

We developed four new score models using newly found significant prognostic variables,which expanded CURB-65 and A-DROP scores. As NT-proBNP and albumin are not available on admission in some hospitals, we constructed two models for each score. Model 1 consists of eight parameters, i.e., confusion, urea >7 mmol/L, respiration rate ≥30 breaths/min, blood pressure (systolic <90 mmHg or diastolic ≤60 mmHg), age ≥65 years, malignancy, heart rate ≥100/min, and lactate >1.7 mmol/L. Model 2 consists of 10 parameters, i.e., confusion, urea >7 mmol/L, respiratory rate ≥30 breaths/min, blood pressure (systolic <90 mmHg or diastolic ≤60 mmHg), age ≥65 years, malignancy, heart rate ≥100/min, albumin ≤3.09 g/dL, lactate >1.7 mmol/L, and NT-ProBNP >500 pg/mL. Model 3 consists of eight parameters, i.e., age ≥70 years for males or ≥75 years for females, blood urea nitrogen ≥21 mg/dL or dehydration, oxyhemoglobin saturation measured by pulse oximetry ≤90% or partial pressure of oxygen in arterial blood ≤60 mmHg, confusion, systolic blood pressure ≤90 mmHg, malignancy, heart rate ≥100/min, and lactate >1.7 mmol/L. Model 4 is composed of 10 parameters, i.e., age ≥70 years for males or ≥75 years for females, blood urea nitrogen ≥21 mg/dL or dehydration, oxyhemoglobin saturation measured by pulse oximetry ≤90% or partial pressure of oxygen in arterial blood ≤60 mmHg, confusion, systolic blood pressure ≤90 mmHg, malignancy, heart rate ≥100/min, albumin ≤3.09 g/dL, lactate >1.7 mmol/L, and NT-ProBNP >500 pg/mL. Respiratory rate ≥24/min was not added in the new score because CURB-65 and A-DROP scores already included respiratory parameters.

The predictive value of expanded CURB-65 score for prediction of 28-day mortality was superior (Model 1 AUC = 0.784, 95% CI: 0.740–0.828, Model 2 AUC = 0.821, 95% CI: 0.781–0.861) to PSI (AUC = 0.735, 95% CI: 0.686–0.784). The predictive value of expanded A-DROP score for prediction of 28-day mortality was also superior (Model 3 AUC = 0.805, 95% CI: 0.761–0.848, Model 4 AUC = 0.834, 95% CI: 0.794–0.874) to other scoring systems, such as PSI (AUC = 0.735, 95% CI: 0.686–0.784), CURB-65 score (AUC = 0.701, 95% CI: 0.648–0.754), and A-DROP score (AUC = 0.730, 95% CI: 0.678–0.782). Expanded A-DROP score (model 4) showed the highest predictive value among the four models. Validation of the expanded A-DROP score (model 4) using bootstrap resampling methods yielded an AUC of 0.833.

## Discussion

Among 1,031 patients with CAP, the 28-day mortality rate was 9.3% in this study. AUCs from the ROC analysis for the prediction of 28-day mortality were 0.735 (95% CI: 0.686–0.784), 0.701 (95% CI: 0.648–0.754), and 0.730 (95% CI: 0.678–0.782) for the PSI, CURB-65, and A-DROP scores, respectively. We showed that the presence of malignancy as a comorbidity, tachypnea, tachycardia, low albumin level, high lactate level, and high NT-ProBNP level were independent predictors of poor prognosis in patients with CAP. In addition, we proposed a new pneumonia severity score using newly identified prognostic variables. The expanded A-DROP score, based on one comorbidity (malignancy), tachycardia, and three laboratory findings (albumin, lactate, and NT-ProBNP levels), predicted mortality with a larger AUC (0.834) than that for the PSI. To our knowledge, this is the largest study to evaluate the usefulness of pneumonia severity score systems for the prediction of mortality in South Korean populations.

The mortality rate in our study was higher than in previous studies performed in the USA and Europe^12^. The number of patients with PSI risk class IV–V was higher in our study compared to those performed in the USA and Europe, which can explain the higher mortality in our cohort.

The ROC scores for CURB-65 and A-DROP in this study were lower than in some previous studies^7,13^. However, many studies showed lower ROC scores for CURB-65 and A-DROP than in our study^14–17^. The reasons for this difference are not clear. Further multicenter studies in larger numbers of patients are needed to improve the efficacy of these scores.

Five important factors improved the performance of the A-DROP score: the presence of malignancy, tachycardia, hypoalbuminemia, increased blood lactate level, and increased NT-ProBNP level. Malignancy is a well-established prognostic factor for CAP, worth 30 points in the PSI^3^. Ito *et al*.^18^ reported that the presence of malignancy was a prognostic factor for hospitalized patients with CAP aged >15 years. Similarly, we found that malignancy was a poor prognostic factor for hospitalized patients with CAP aged >18 years.

Tachycardia is a known prognostic factor for CAP. It is included in the PSI (heart rate ≥125 beats/min, 10 points)^3^ and in the Acute Physiology and Chronic Health Evaluation II score^19^. Several studies have shown that tachycardia is a prognostic factor for CAP^20,21^. However, as heart rate cut-off values have varied among studies, further studies are needed to confirm the usefulness of this factor.

Among laboratory parameters, we found that the albumin, lactate, and NT-ProBNP levels were significant prognostic factors for CAP. Albumin is synthesized in the liver using the amino acids in hepatocytes. Thus, decreased liver function and malnutrition may result in hypoalbuminemia. An imbalance between intravascular and extravascular albumin levels may also result in hypoalbuminemia. Kim *et al*.^22^ reported that patients aged >18 years with debilitating conditions and aspiration pneumonia were assigned more frequently to the hypoalbuminemia group than to the non-hypoalbuminemia group. Previous studies have shown that a low initial serum albumin concentration is an independent risk factor for mortality in patients with CAP^13,18,22^. In our study, hypoalbuminemia had the highest OR for mortality prediction. Malnutrition or underlying disease might influence the mortality of CAP.

The lactate level has been used widely in critically ill patients to assess perfusion status, organ dysfunction, treatment response, and prognosis^23^. In patients with pneumonia, hyperlactatemia is associated with mortality, hospitalization, and intensive care unit admission. The addition of the lactate level significantly improved the prognostic value of the CURB-65 score for CAP mortality prediction in previous studies^15,24^. NT-ProBNP is used in the assessment of cardiac dysfunction. It is secreted in response to excessive stretching of cardiomyocytes and regulates natriuresis, body fluid volume, vascular pressure, and electrolyte balance^25,26^. Some studies have suggested that increased BNP levels in patients with CAP are related to the inflammatory response and local hypoxia in the pulmonary circulatory system^27^. NT-ProBNP was a strong predictor of mortality in hospitalized patients with CAP, with a performance in predicting mortality comparable to that of the PSI and CURB-65 scores^28,29^.

**Figure 2:**
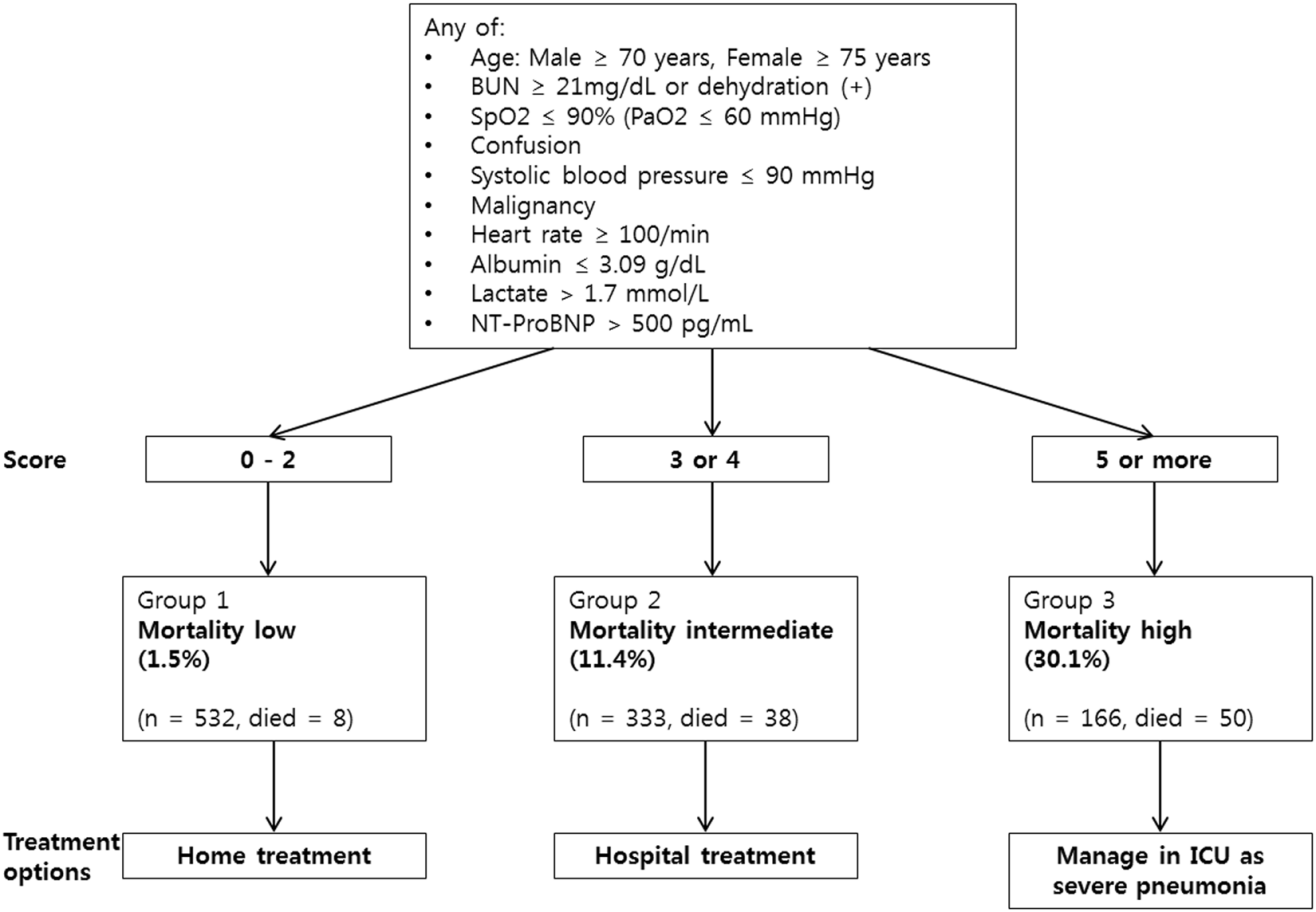
Clinical severity assessment in a patient with CAP. CAP: community-acquired pneumonia; BUN: blood urea nitrogen; SpO_2_: oxygen saturation; PaO_2_: partial pressure of arterial oxygen; NT-ProBNP: N-terminal pro-brain natriuretic peptide; ICU: intensive care unit.

The expanded A-DROP score, which employs 10 significant risk factors to predict CAP severity, has greater predictive value than do preexisting severity scores. As NT-proBNP and albumin are not available on admission in some hospitals, we constructed two models. Not only model 4 (10 parameters) but also model 3 (8 parameters) showed higher predictive value compared to pre-existing severity scores. This finding has several significant implications. First, the score decreased the relative effects of age and comorbidities and removed the need for use radiographic findings in the calculation, as compared to PSI. PSI tends to weight age and comorbidities heavily. The expanded A-DROP score significantly improved prediction of high-risk patients by alleviating the weight of age and comorbidities in the calculation. In addition, the score includes several significant biomarkers that are not included in other severity scores. After addressing weaknesses in the preexisting score, the expanded A-DROP score showed greatly improved predictive value (AUC = 0.834). We found similar results (AUC = 0.833) with bootstrap validation. Second, the score can be used in real practice; it is a simpler (10-variable) alternative to the PSI (20-variable) for the prediction of short-term mortality in patients with CAP. The results for the expanded A-DROP score were superior to those for the PSI. In this study, patients with scores of 0, 1, and 2 were considered to be at low risk (1.5%) of mortality, with the possibility of management on a hospital outpatient basis. Patients with scores of 3 and 4 were regarded to be at intermediate risk (11.4%) of mortality, such that hospitalization should be considered. Patients with scores ≥5 were considered to be at high risk (30.1%) of mortality, and initial care in an intensive care unit should be considered (Fig. 2). However, we emphasize that the applicability of the expanded A-DROP score can only be determined by external validation in different cohorts of CAP patients. We hope that other groups will validate the accuracy of this score for predicting mortality in other patient populations.

This study had several limitations. First, as no external validation was conducted in this study, the applicability of the expanded A-DROP score in clinical practice requires further study. Second, it was retrospective and conducted at a single center in South Korea, which had only hospitalized patients. Thus, this new scoring system cannot be applied to outpatients. Third, medical records regarding restriction of treatment escalation, such as do-not-resuscitate orders, were insufficient. Thus, the influence of such restriction on mortality was not considered. Fourth, selection bias could not be avoided as we did not use population-based data and the severity of patients’ conditions may differ among tertiary care hospitals in the same area.

In conclusion, five clinical factors – the presence of malignancy, tachycardia, hypoalbuminemia, increased blood lactate level, and increased NT-ProBNP level – are independent predictors of 28-day mortality in patients with CAP. By adding these predictors to the original A-DROP score, we developed a simpler and more accurate scoring system for the prediction of CAP severity in hospitalized patients.

## References

[1] Mandell LA (2007). Infectious Diseases Society of America/American Thoracic Society consensus guidelines on the management of community-acquired pneumonia in adults. Clinical Infectious Diseases: an Official Publication of the Infectious Diseases Society of America.

[2] Shin HY (2016). Cause-of-death statistics in the Republic of Korea, 2014. J Korean Med Assoc.

[3] Fine MJ (1997). A prediction rule to identify low-risk patients with community-acquired pneumonia. The New England Journal of Medicine.

[4] Lim WS (2003). Defining community acquired pneumonia severity on presentation to hospital: an international derivation and validation study. Thorax.

[5] Miyashita N, Matsushima T, Oka M, Japanese Respiratory S (2006). The JRS guidelines for the management of community-acquired pneumonia in adults: an update and new recommendations. Intern Med.

[6] Usui K, Tanaka Y, Noda H, Ishihara T (2009). [Comparison of three prediction rules for prognosis in community acquired pneumonia: Pneumonia Severity Index (PSI), CURB-65, and A-DROP]. Nihon Kokyuki Gakkai zasshi = The Journal of the Japanese Respiratory Society.

[7] Shindo Y (2008). Comparison of severity scoring systems A-DROP and CURB-65 for community-acquired pneumonia. Respirology.

[8] Carratala J (2005). Outpatient care compared with hospitalization for community-acquired pneumonia: a randomized trial in low-risk patients. Annals of Internal Medicine.

[9] Kalil AC (2016). Management of Adults With Hospital-acquired and Ventilator-associated Pneumonia: 2016 Clinical Practice Guidelines by the Infectious Diseases Society of America and the American Thoracic Society. Clinical Infectious Diseases: an Official Publication of the Infectious Diseases Society of America.

[10] Ahn JH (2017). Clinical characteristics and prognostic risk factors of healthcare-associated pneumonia in a Korean tertiary teaching hospital. Medicine.

[11] Steyerberg EW (2001). Internal validation of predictive models: efficiency of some procedures for logistic regression analysis. Journal of Clinical Epidemiology.

[12] Arnold FW, Wiemken TL, Peyrani P, Ramirez JA, Brock GN (2013). Mortality differences among hospitalized patients with community-acquired pneumonia in three world regions: results from the Community-Acquired Pneumonia Organization (CAPO) International Cohort Study. Respiratory Medicine.

[13] Liu JL (2016). Expanded CURB-65: a new score system predicts severity of community-acquired pneumonia with superior efficiency. Scientific Reports.

[14] Jo S (2016). Validation of modified early warning score using serum lactate level in community-acquired pneumonia patients. The National Early Warning Score-Lactate score. The American Journal of Emergency Medicine.

[15] Chen YX, Li CS (2015). Lactate on emergency department arrival as a predictor of mortality and site-of-care in pneumonia patients: a cohort study. Thorax.

[16] Pflug MA (2015). Short-term mortality of adult inpatients with community-acquired pneumonia: external validation of a modified CURB-65 score. Postgraduate Medical Journal.

[17] Nullmann H (2014). External validation of the CURSI criteria (confusion, urea, respiratory rate and shock index) in adults hospitalised for community-acquired pneumonia. BMC Infectious Diseases.

[18] Ito A (2017). Prognostic factors in hospitalized community-acquired pneumonia: a retrospective study of a prospective observational cohort. BMC Pulmonary Medicine.

[19] Knaus WA, Draper EA, Wagner DP, Zimmerman JE (1985). APACHE II: a severity of disease classification system. Critical Care Medicine.

[20] Braun E, Kheir J, Mashiach T, Naffaa M, Azzam ZS (2014). Is elevated red cell distribution width a prognostic predictor in adult patients with community acquired pneumonia?. BMC Infectious Diseases.

[21] Kolditz M (2015). Community-acquired pneumonia as medical emergency: predictors of early deterioration. Thorax.

[22] Kim Hyosun, Jo Sion, Lee Jae Baek, Jin Youngho, Jeong Taeoh, Yoon Jaechol, Lee Jeong Moon, Park Boyoung (2018). Diagnostic performance of initial serum albumin level for predicting in-hospital mortality among aspiration pneumonia patients. The American Journal of Emergency Medicine.

[23] Singer M (2016). The Third International Consensus Definitions for Sepsis and SepticShock (Sepsis-3). Jama.

[24] Frenzen F.S., Kutschan U., Meiswinkel N., Schulte-Hubbert B., Ewig S., Kolditz M. (2018). Admission lactate predicts poor prognosis independently of the CRB/CURB-65 scores in community-acquired pneumonia. Clinical Microbiology and Infection.

[25] Hall C (2004). Essential biochemistry and physiology of (NT-pro)BNP. European Journal of Heart Failure.

[26] Brueckmann M (2005). Prognostic value of plasma N-terminal pro-brain natriuretic peptide in patients with severe sepsis. Circulation.

[27] Li J, Ye H, Zhao L (2015). B-type natriuretic peptide in predicting the severity of community-acquired pneumonia. World Journal of Emergency Medicine.

[28] Jeong KY (2011). Prognostic value of N-terminal pro-brain natriuretic peptide in hospitalised patients with community-acquired pneumonia. Emergency Medicine Journal: EMJ.

[29] Chang CL (2013). Biomarkers of cardiac dysfunction and mortality from community-acquired pneumonia in adults. PloS one.

